# Supplementary material for: IFN-γ selectively suppresses a subset of TLR4-activated genes and enhancers to potentiate macrophage activation
Source: Nat Commun. 2019 Jul 25;10:3320. doi: 10.1038/s41467-019-11147-3 (PMC6658531; doi:10.1038/s41467-019-11147-3)
Supplement: Supplementary file 1 — Supplementary Information [file 41467_2019_11147_MOESM1_ESM.pdf]

IFN-γ selectively suppresses a subset of TLR4-activated genes and enhancers to potentiate macrophage activation

Kyuho K, Bachu M et al.

Supplementary Information

Supplementary Fig. 1.

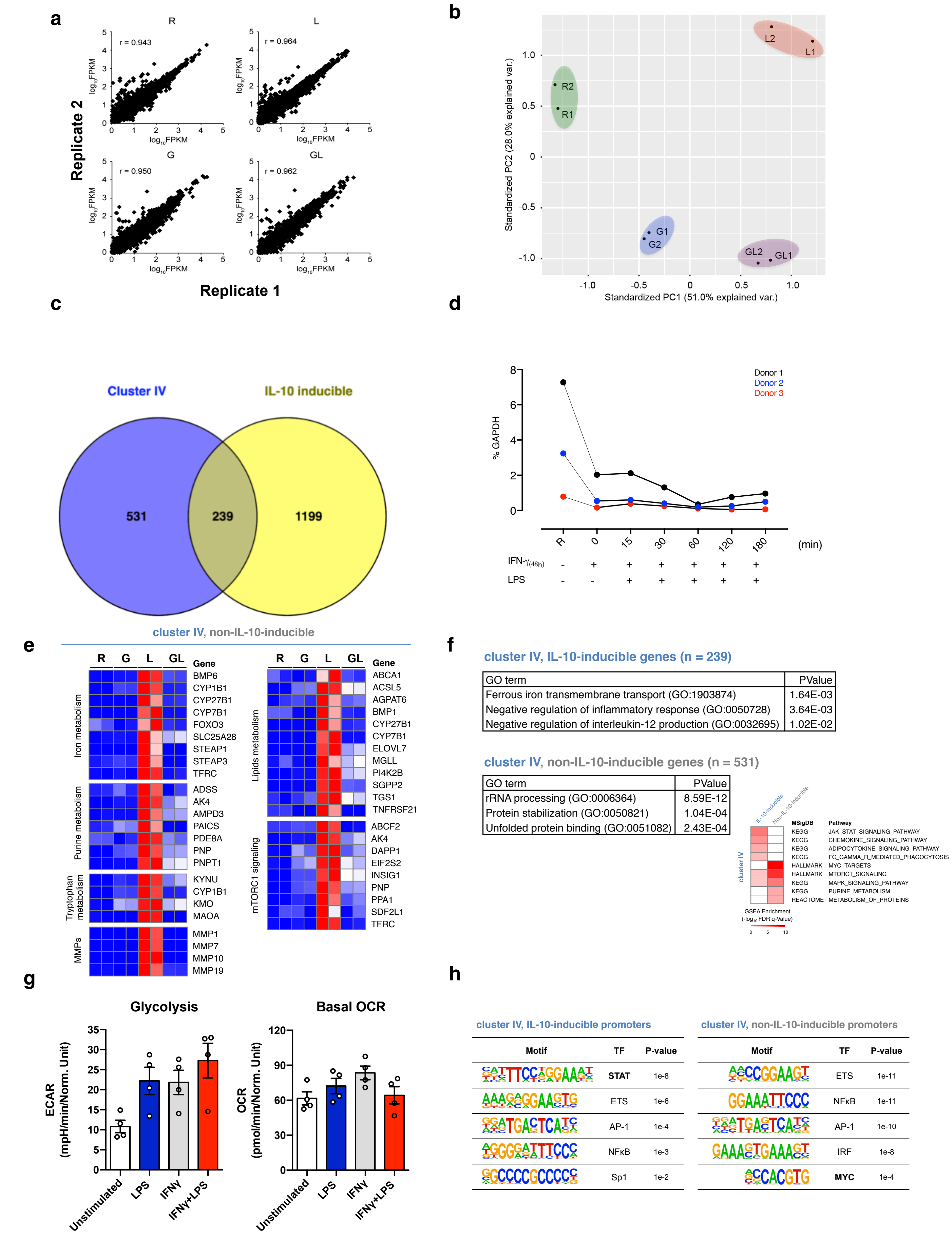

Transcriptomic profiling of IFN-γ-primed LPS-stimulated macrophages. (a) Correlation of RNA-seq replicates. (b) Principle Component Analysis (PCA) for RNA-seq replicates among four conditions. (c) Venn diagram showing number of genes in cluster IV (n = 531, blue), genes induced by IL-10 alone (n = 1199, yellow), and the overlap between cluster IV and IL-10-inducible genes (n = 239). (d) Kinetic analysis of *IL10* mRNA expression in CD14^{+} monocytes cultured with M-CSF (20 ng/ml) + IFN- γ (100 U/ml) for 48 hours followed by exposure to LPS (10 ng/ml) for various times 15, 30, 60, 120 and 180 min. The *IL10* expression data are plotted as percentage of GAPDH expression for each donor (n=3). (e) Heatmap showing the relative expression of examples of cluster IV genes that are not inducible by IL-10. Distinct biological functions showed on the left. (f) Gene ontology (GO) analysis using DAVID 6.8. Heat map showing the FDR q-value significance of GSEA enrichment for the two classes of cluster IV genes classified in (c). (g) Human CD14^{+} monocytes were cultured with M-CSF (20 ng/ml) and IFN-γ (100 U/ml) for two days and then were stimulated with or without LPS (50 ng/ml). Extracellular acidification rate (ECAR, a surrogate marker of glycolysis, left panel) and oxygen consumption rate (OCR, right panel) were measured by Seahorse assay with a glycolysis stress test kit. Data are shown as mean ± SEM from four independent donors. (h) Known motif analysis of promoters. Data is representative of two experiments.

Supplementary Fig. 2.

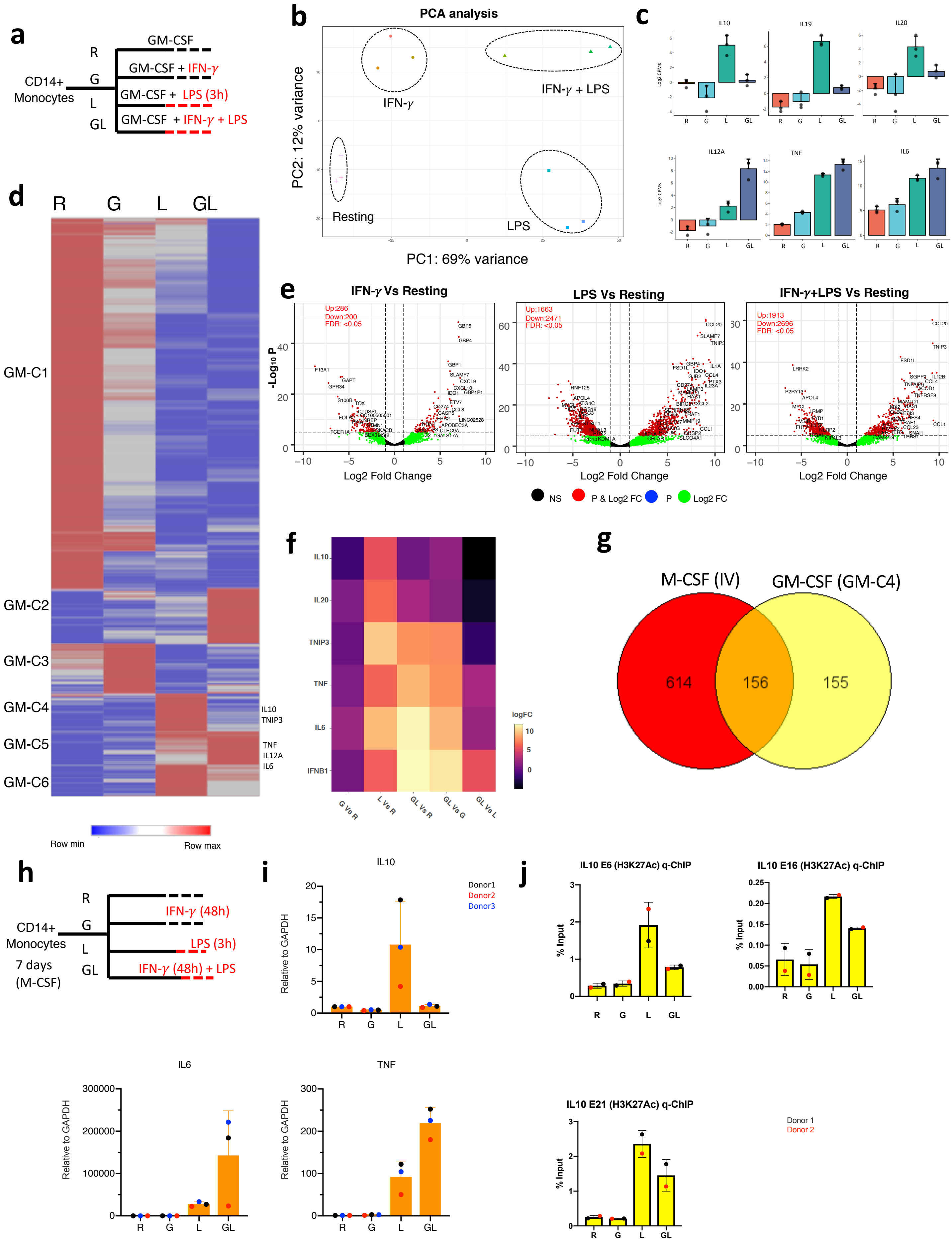

Transcriptomic profiling of macrophages differentiated by prolonged culture with GM-CSF or M-CSF prior to IFN-γ-treatment and LPS-stimulation. (a) Experimental design: human CD14+ monocyte-derived macrophages cultured for 5 days in the presence of 20 ng/ml of GM-CSF underwent one of four different combinations of priming for 48 hr with IFN-γ before stimulation with LPS (or not) for 3 hr: without priming or stimulation (R); primed with IFN-γ without LPS stimulation (G); LPS stimulation only with no priming (L); or primed with IFN-γ and stimulated with LPS (GL). (b) Principal component analysis of RNA-seq data from three independent donors showing the effect of various treatments (PC1:69% variance) on RNA expression (c) Normalized expression (log2CPM) of representative genes in clusters GM-C4 (top) and GM-C5 (bottom); clusters are defined in (d). Each dot in the bar plot represents one donor and error bars represent standard deviation. (d) K-means (K = 6) clustering of 5,370 differentially expressed genes in any pairwise comparison among four conditions. Clusters are indicated on the left. (e) Volcano plots showing differentially regulated genes. NS (black) = not significant; P & Log2FC (red) = p-adjusted value < 0.05 and >2-fold change, P (blue) = p-adjusted value =< 0.05; Log2FC (green) = >2-fold change but not significant. (f) Heatmap of log2FC across five combinations of select GM-C4 (*IL10*, *IL20* & *TNIP3*) and GM-C5 (*TNF*, *IL6* and *IFNB1*) genes. (g) Venn diagram showing overlap of genes in cluster IV (C IV) in cells cultured with M-CSF (C IV=770) and GM-CSF (GMC4=310). (h) Experimental design: human CD14+ monocyte-derived macrophages cultured in the presence of 20 ng/ml of M-CSF for 7 days underwent one of four different combinations of priming for 48 hr with IFN-γ before stimulation with LPS (or not) for 3 hr: without priming or stimulation (R); primed with IFN-γ without LPS stimulation (G); LPS stimulation only with no priming (L); or primed with IFN-γ and stimulated with LPS (GL). (i) Analysis of RNA expression of *IL10*, *IL6* and *TNF* in prolonged macrophage cultures upon treatment with different combinations of IFN-γ and LPS as shown in (h). The plotted q-PCR data are from three independent donors (black, red and blue). Error bars represent standard deviation (j) ChIP q-PCR analysis of H3K27Ac upon various treatments as shown in (h). E6 enhancer is located 6kb downstream from the transcription start site (TSS) of *IL10* and E16 and E21 enhancers are located 16 and 21 kb, respectively, upstream from the TSS. The data are from two independent donors (black, red) and error bars represent standard deviation.

Supplementary Fig. 3.

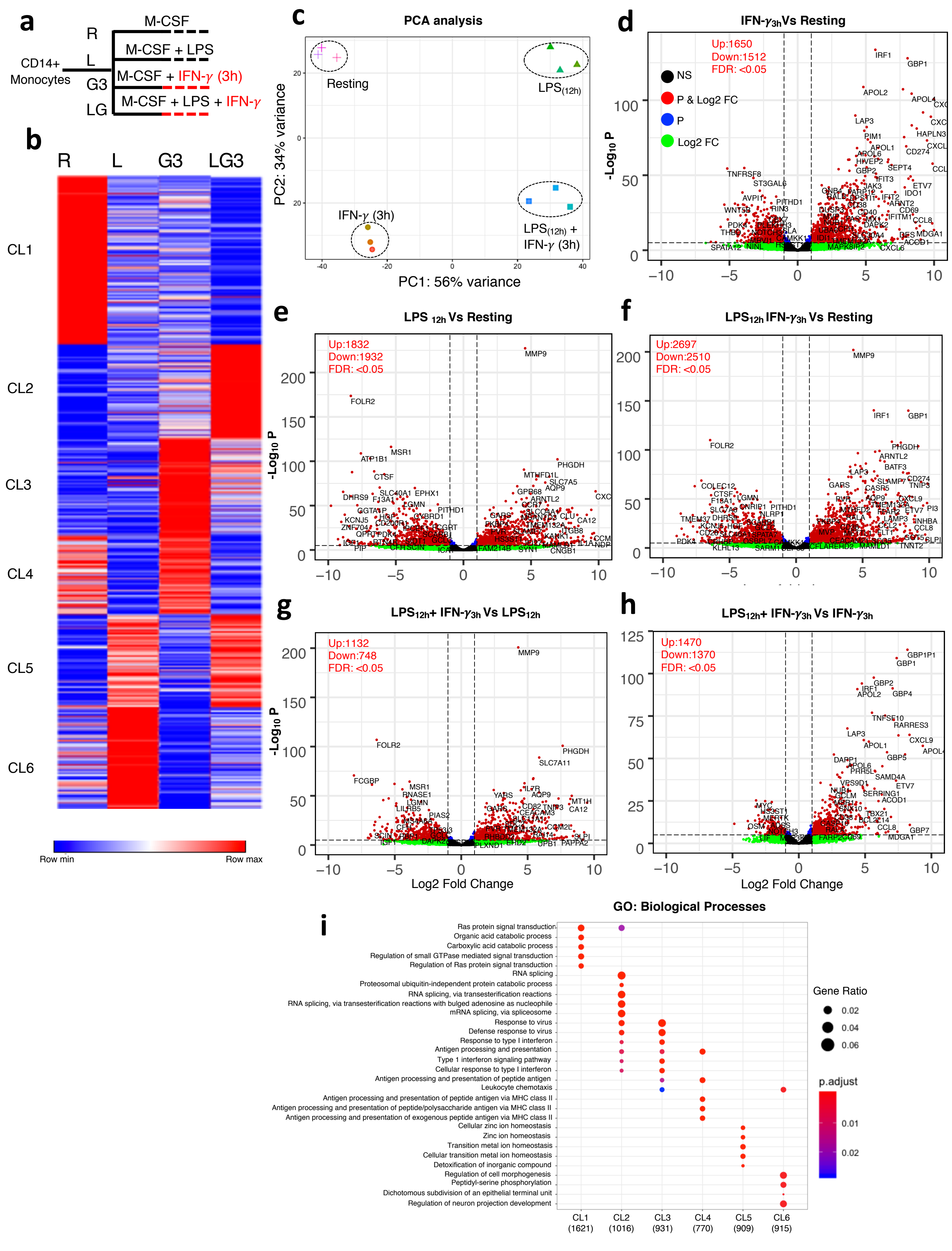

Transcriptomic profiling of macrophages upon LPS treatment followed by IFN-γ stimulation. (a) Experimental design: human CD14[+] monocyte-derived macrophages cultured in the presence of 20 ng/ml of M-CSF underwent one of four different combinations of treatment with LPS (0.1 ng/ml) overnight before stimulation with IFN-γ (or not) for 3 hr: without LPS or IFN-γ treatment (R); treated with LPS without IFN-γ stimulation (L); IFN-γ stimulation only with no LPS (G3); or treated with LPS and stimulated with IFN-γ (LG3). (b) K-means (K = 6) clustering of 8,047 differentially expressed genes in any pairwise comparison among four conditions. Clusters are indicated on the left. (c) Principal component analysis of RNA-seq data from three independent donors showing the effect of various treatments (PC1:56% variance) on RNA expression. (d-h) Volcano plots showing differentially regulated genes, NS (black) = not significant; P & Log2FC (red) = p-adjusted value < 0.05 and >2-fold change, P (blue) = p-adjusted value =< 0.05; Log2FC (green) = >2-fold change but not significant. (i) Gene ontology analysis of biological processes on various gene clusters using enrichGO function from clusterProfiler (Bioconductor version: Release (3.8) package). The dot plot shows enrichment of GO terms for various biological processes. The x-axis denotes the number of genes in each of the gene clusters, dot size represents gene ratio and color represents adjusted p-value for each GO term.

Supplementary Fig. 4.

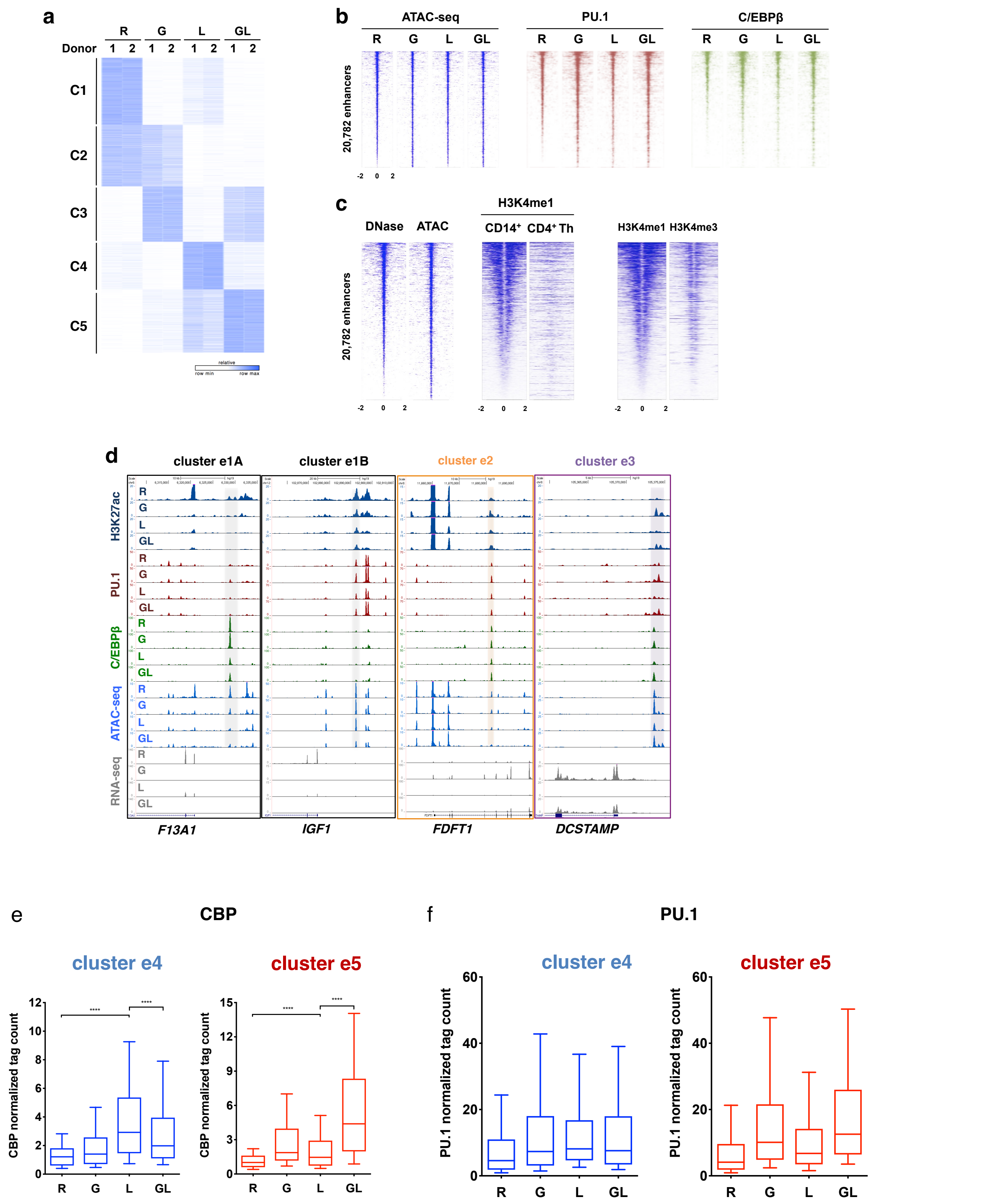

IFN-γ regulates TLR4-activated enhancer landscapes. (a) K-means clustering (K = 5) of H3K27ac ChIP-seq intensity in open chromatin regions (ATAC-seq peaks) that were bound by PU.1 or C/EBPβ. (b) Heatmaps of ATAC-seq, PU.1, and C/EBPβ ChIP-seq signals at 20,782 enhancers differentially regulated at the H3K27-Ac level. (c) Left panels show congruence of ATAC-seq peaks with DNase-seq peaks from ENCODE project. Heatmaps of normalized tag densities for DNase-seq (CD14$^+$ monocytes from ENCODE, GSM1024791) and ATAC-seq (monocyte-derived macrophages used in this project) at the 20,782 enhancers defined and analyzed in this project. Middle panels show heatmaps of normalized tag densities for H3K4me1 ChIP-seq from CD14$^+$ monocytes (left, GSM1102793) and CD4$^+$ T cells (right, GSM1220567) at the 20,782 enhancers. Right panels show heatmaps of normalized tag densities for H3K4me1 ChIP-seq from CD14$^+$ monocytes (left) and H3K4me3 ChIP-seq from CD14$^+$ monocytes (right, GSM945225) at the 20,782 enhancers. Four-kilobase windows are shown centered at the midpoints of the ATAC-seq peak. (d) Representative UCSC Genome Browser tracks displaying normalized tag-density profiles at enhancers of *F13A1, IGF1, FDFT1,* and *DCSTAMP* in the four indicated conditions. Boxes enclose cluster e1A, e1B, e2, and e3 enhancers. (e) Boxplots depicting normalized tag counts of CBP at e4 and e5 enhancer clusters. (f) Boxplots depicting normalized tag counts of PU.1 at e4 and e5 enhancer clusters.

Supplementary Fig. 5.

a

Cluster e1A, FDR < 0.05 (n = 1,610)

Motif	TF	P-value
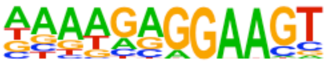	PU.1	1e-443
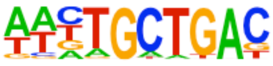	MAF	1e-69
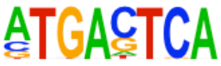	AP-1	1e-55
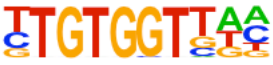	RUNX	1e-26
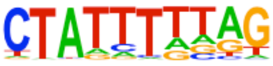	MEF2	1e-24
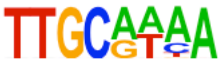	C/EBP	1e-18

Cluster e1B, FDR < 0.05 (n = 1,146)

Motif	TF	P-value
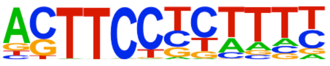	PU.1	1e-310
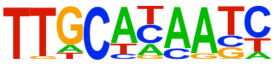	C/EBP	1e-36
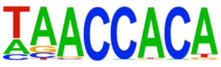	RUNX	1e-21
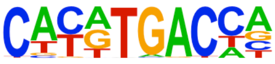	MITF	1e-20
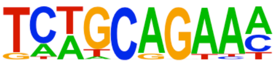	MAF	1e-16
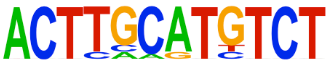	IRF	1e-13

b

Cluster e2, FDR < 0.05 (n = 668)

Motif	TF	P-value
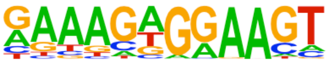	PU.1	1e-127
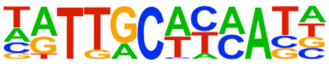	C/EBP	1e-29
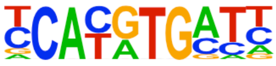	TFE	1e-26
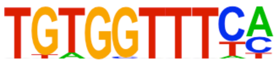	RUNX	1e-14

Cluster e3, FDR < 0.05 (n = 866)

Motif	TF	P-value
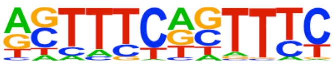	IRF	1e-160
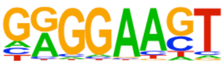	PU.1	1e-62
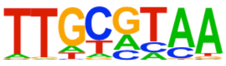	C/EBP	1e-30
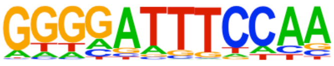	NFκB	1e-23
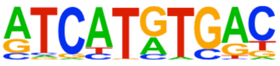	MITF	1e-21

c

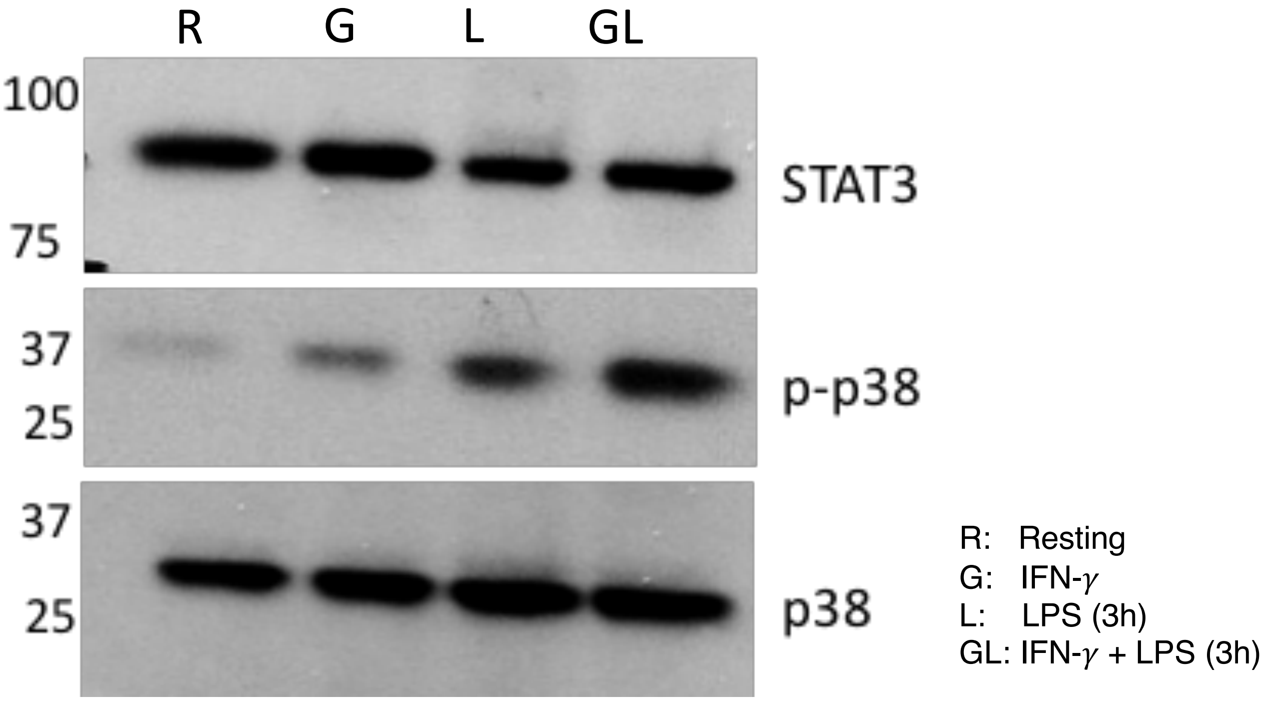

Distinct motif enrichment in different enhancer clusters. (a) The most significantly enriched transcription factor (TF) motifs identified by *de novo* motif analysis using HOMER in cluster e1A (left) and cluster e1B (right) enhancers. (b) The most significantly enriched transcription factor (TF) motifs identified by *de novo* motif analysis using HOMER at cluster e2 (left) and cluster e3 (right) enhancers. (c) STAT3 protein levels was measured by western blot. Whole cell lysates from human CD14^{+} monocytes exposed to various stimuli (R: Resting, G: IFN- γ, L:LPS (3h), GL: IFN- γ and LPS (3h)) were subjected to immunoblot analysis using STAT3, phospho-p38 and p38 antibodies.

Supplementary Fig. 6.

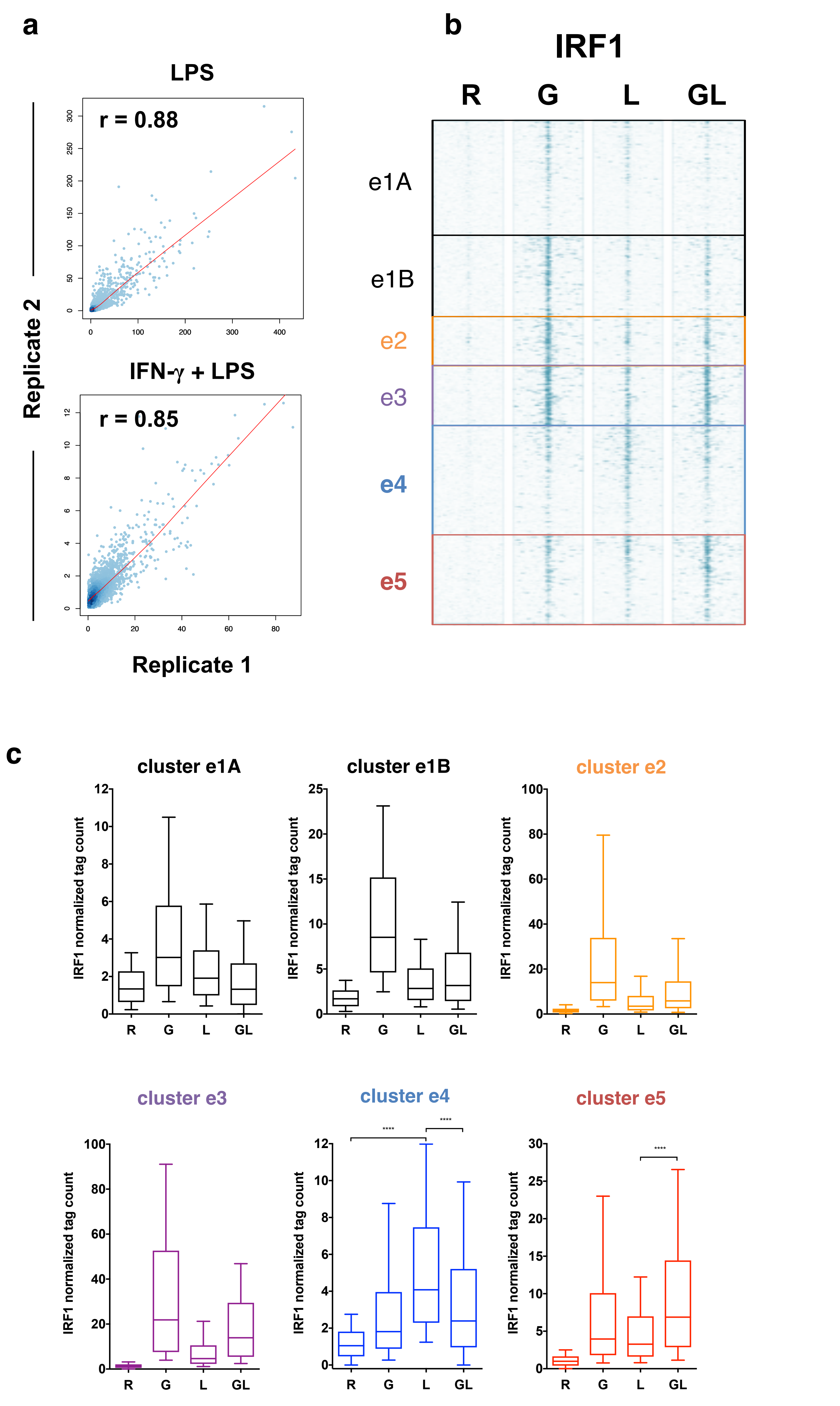

Asymmetric TF binding at each enhancer cluster. (a) Correlation of STAT3 ChIP-seq replicates in LPS-stimulated (top) and IFN-γ-primed LPS-stimulated macrophages (bottom). (b) Heatmaps showing IRF1 ChIP-seq signals at each enhancer cluster defined in Fig. 2a. (c) The boxplots indicate IRF1 normalized tag counts at each enhancer cluster. ****$p < 0.0001$, paired-samples Wilcoxon signed-rank test.

Supplementary Fig. 7.

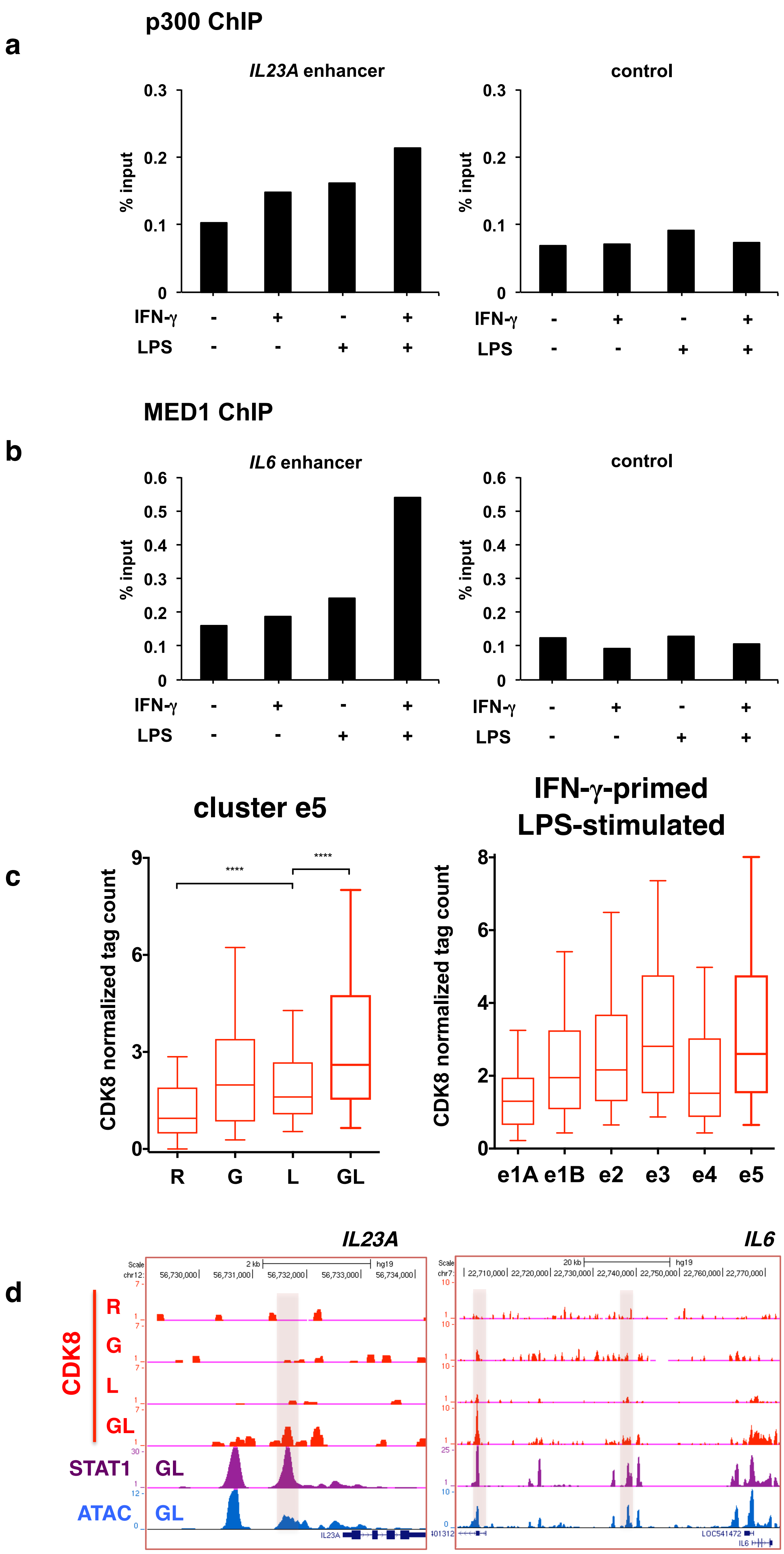

IFN-γ priming increases LPS-induced binding of p300, MED1 and CDK8 at e5 enhancers. (a) ChIP-qPCR analysis of p300 occupancy at the e5 enhancer of *IL23A*. (b) ChIP-qPCR of MED1 at the e5 enhancer of *IL6*. Data are representative of three independent experiments. (c) The boxplot (left) indicates CDK8 normalized tag counts at e5 enhancers in the four indicated conditions. ****$p < 0.0001$, paired-samples Wilcoxon signed-rank test. The boxplot (right) indicates CDK8 normalized tag counts at each enhancer cluster in IFN-γ-primed LPS-stimulated macrophages. (d) Representative UCSC Genome Browser tracks displaying normalized tag-density profiles at e5 enhancers of *IL23A* and *IL6* in the four indicated conditions (CDK8) and the LPS-stimulated condition (STAT1 ChIP-seq and ATAC-seq).

Supplementary Fig. 8.

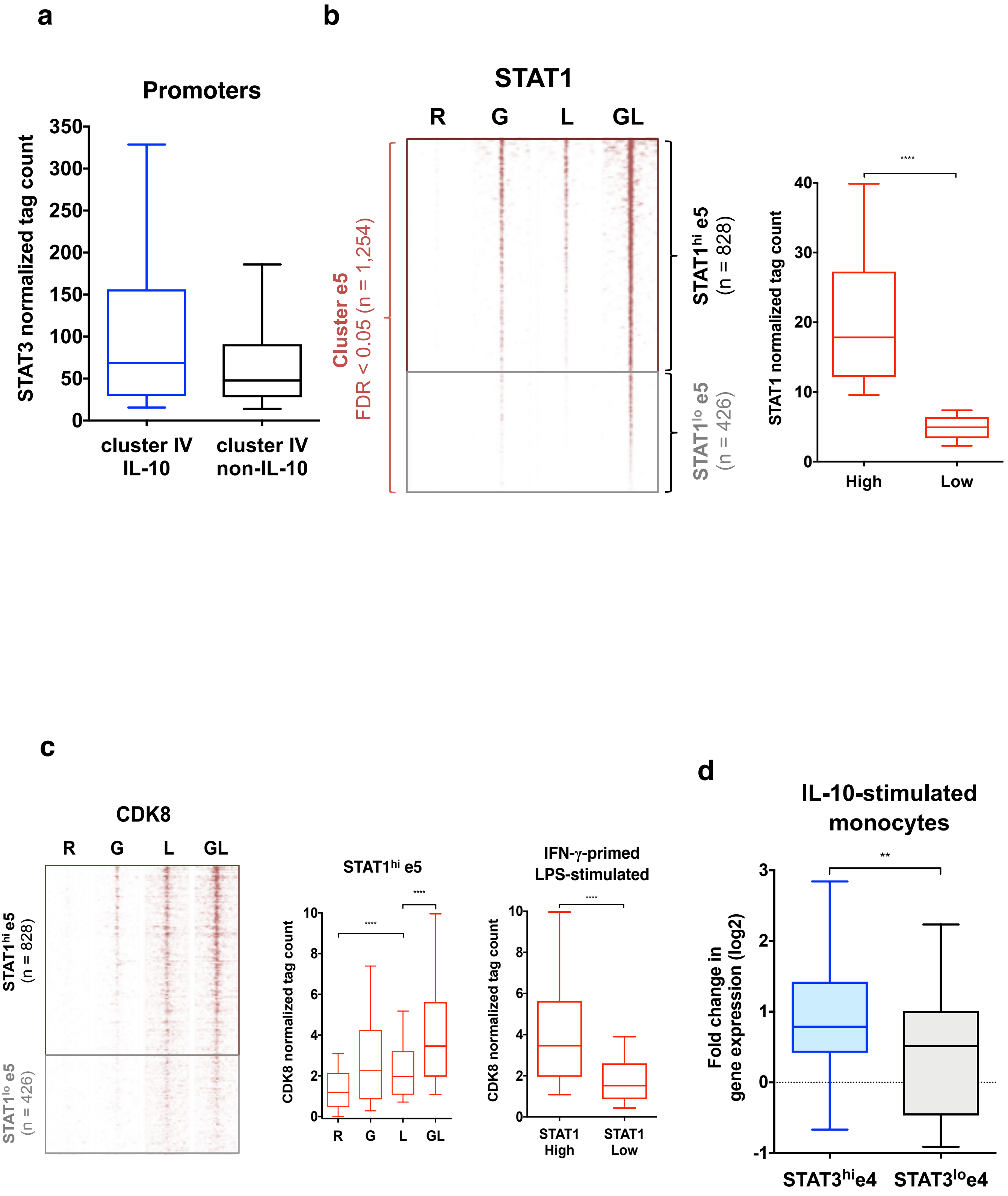

e5 enhancers subdivide into two groups based on coordinate STAT1 and CDK8 occupancy. (a) The boxplot indicates normalized tag counts at promoters of IL-10-inducible cluster IV genes (blue) and non-IL-10-inducible cluster IV genes (black). (b) Heatmap of STAT1 ChIP-seq signals at cluster e5 enhancers in the four indicated conditions. Enhancers were separated into two subsets: $STAT1^{hi}e5$ (n = 828) and $STAT1^{lo}e5$ (n = 426) based upon a log_2 normalized tag count cutoff od 3). The boxplot (right) indicates normalized tag counts at $STAT1^{hi}e5$ and $STAT1^{lo}e5$ enhancers. (c) Heatmaps of CDK8 ChIP-seq signals at the two subsets of e5 enhancers (defined in b). The boxplots indicate normalized tag counts at $STAT1^{hi}e5$ enhancers (left) and at $STAT1^{hi}e5$ and $STAT1^{lo}e5$ enhancers in IFN-γ-primed LPS-stimulated macrophages (right). ****p < 0.0001, paired-samples Wilcoxon signed-rank test. (d) Boxplots of the change in gene expression after IL-10 stimulation of macrophages of the differentially expressed genes nearest (within 100 kb) to $STAT3^{hi}e4$ (blue) or $STAT3^{lo}e4$ (black) enhancers. **p = 0.0032 by Welch's t test.

Supplementary Fig. 9.

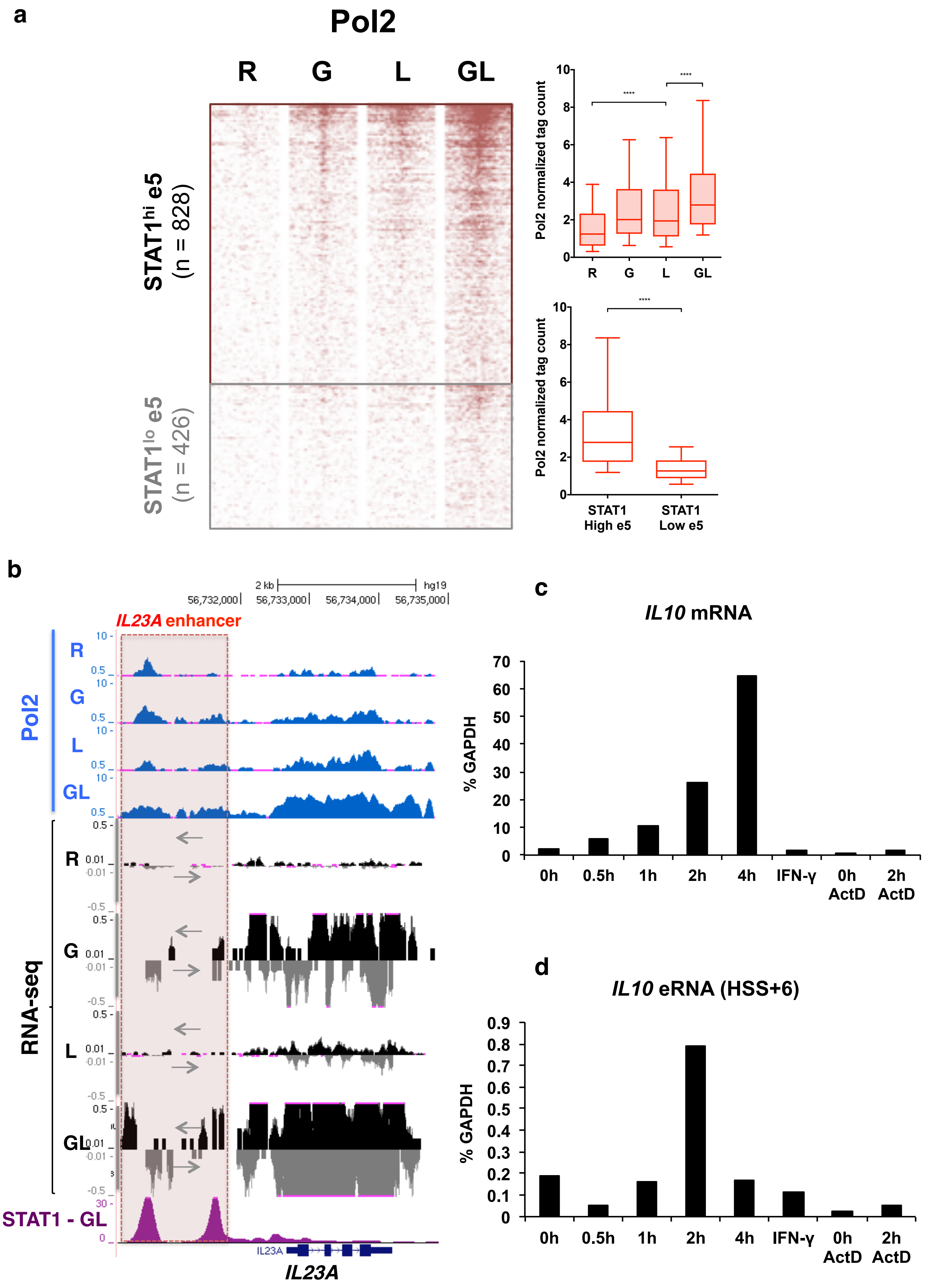

IFN-γ-mediated functional activation of STAT1-bound e5 enhancers. (a) Heatmaps of Pol II ChIP-seq signals at STAT1^{hi}e5 and STAT1^{lo}e5 enhancers (defined in Supplementary Fig. 6b) in the four indicated conditions. The boxplots indicate normalized tag counts at STAT1^{hi}e5 enhancers (top) and at STAT1^{hi}e5 and STAT1^{lo}e5 enhancers in IFN-γ-primed LPS-stimulated macrophages (bottom). ****$p < 0.0001$, paired-samples Wilcoxon signed-rank test. (b) Representative Genome Browser tracks showing RNA polymerase II (Pol II) occupancy, strand-specific RNA transcripts, and STAT1 occupancy (IFN-γ + LPS condition) at enhancers of *IL23A*. Box encloses enhancer of *IL23A*. (c) RT-qPCR analysis of *IL10* mRNA expression in macrophages cultured without or with IFN-γ for 48 hr and then stimulated with LPS. 5 μg/mL actinomycin D (Act D) was added 1 hr prior to harvesting. (d) RT-qPCR analysis of *IL10* eRNA (HSS+6) expression in macrophages cultured without or with IFN-γ for 48 hr and then stimulated with LPS for the indicated time course. 5 μg/mL actinomycin D (ActD) was added 1 hr prior to harvesting. Data are representative of two independent experiments.

Supplementary Table 1.

Primers for expression of mRNA or eRNA and ChIP-qPCR.

hIL10_F	GACTTTAAGGGTTACCTGGGTTG
hIL10_R	TCACATGCGCCTTGATGTCTG
ChIP_control_F	GGTTGTGGTGGTAAGAAGTTGA
ChIP_control_R	TGAAAACACTCCAAGGCAGA
IL10_eRNA_HSS+6_F	TTCTTCAGTTCAGGATGGACAG
IL10_eRNA_HSS+6_R	GCGAAAATTCTGAGATGAAAGG
IL10_eRNA_HSS-16_F	TTCCTTCCTTGTGACATGGTT
IL10_eRNA_HSS-16_R	CATAGAACAGCTGCAAAGAGTCA
IL10+enh6-FP	GGGACTTGTGAACCCTGAAA
IL10+enh6-RP	TTGGGCACATGTCATCAGAA
IL10-enh16-FP	CCTGGGTTTCGGTCTTTCTT
IL10-enh16-RP	CTGAGGAGTCTCTCCATTTCAC
IL10-enh21.1-FP	CCAAGCCTACACAGCTGATAA
IL10-enh21.1-RP	CCATAGCTCTGTTAAGGGACAG
IL23A_eRNA_F	GTCTTGTGAATGCGATTTGC
IL23A_eRNA_R	TTTGTTTCACTTCCTGGTGCT
IL6_eRNA_F	CCATTGCTTTCTAGCTGTGTCA
IL6_eRNA_R	GGAATCAATGTGTAGGCCAAA
