# Supplementary material for: IFN-γ selectively suppresses a subset of TLR4-activated genes and enhancers to potentiate macrophage activation
Source: Nat Commun. 2019 Jul 25;10:3320. doi: 10.1038/s41467-019-11147-3 (PMC6658531; doi:10.1038/s41467-019-11147-3)
Supplement: Supplementary file 2 — Reporting Summary [file 41467_2019_11147_MOESM2_ESM.pdf]

nature research

Corresponding author(s): Kyuho Kang and Lionel B. Ivashkiv

Last updated by author(s): Jun 20, 2019

Reporting Summary

Nature Research wishes to improve the reproducibility of the work that we publish. This form provides structure for consistency and transparency in reporting. For further information on Nature Research policies, see Authors & Referees and the Editorial Policy Checklist.

Statistics

For all statistical analyses, confirm that the following items are present in the figure legend, table legend, main text, or Methods section.

n/a	Confirmed	
☐	☒	The exact sample size (*n*) for each experimental group/condition, given as a discrete number and unit of measurement
☐	☒	A statement on whether measurements were taken from distinct samples or whether the same sample was measured repeatedly
☐	☒	The statistical test(s) used AND whether they are one- or two-sided<br>*Only common tests should be described solely by name; describe more complex techniques in the Methods section.*
☒	☐	A description of all covariates tested
☒	☐	A description of any assumptions or corrections, such as tests of normality and adjustment for multiple comparisons
☐	☒	A full description of the statistical parameters including central tendency (e.g. means) or other basic estimates (e.g. regression coefficient) AND variation (e.g. standard deviation) or associated estimates of uncertainty (e.g. confidence intervals)
☐	☒	For null hypothesis testing, the test statistic (e.g. *F*, *t*, *r*) with confidence intervals, effect sizes, degrees of freedom and *P* value noted<br>*Give P values as exact values whenever suitable.*
☒	☐	For Bayesian analysis, information on the choice of priors and Markov chain Monte Carlo settings
☒	☐	For hierarchical and complex designs, identification of the appropriate level for tests and full reporting of outcomes
☒	☐	Estimates of effect sizes (e.g. Cohen's *d*, Pearson's *r*), indicating how they were calculated

Our web collection on statistics for biologists contains articles on many of the points above.

Software and code

Policy information about availability of computer code

Data collection	GraphPad Prism 7
Data analysis	Provided in methods section. No new algorithms or software were generated for this manuscript.

For manuscripts utilizing custom algorithms or software that are central to the research but not yet described in published literature, software must be made available to editors/reviewers. We strongly encourage code deposition in a community repository (e.g. GitHub). See the Nature Research guidelines for submitting code & software for further information.

Data

Policy information about availability of data

All manuscripts must include a data availability statement. This statement should provide the following information, where applicable:
- Accession codes, unique identifiers, or web links for publicly available datasets
- A list of figures that have associated raw data
- A description of any restrictions on data availability

RNA-seq, ChIP-seq and ATAC-seq data that support the findings of this study have been deposited in GenBank with the GSE120945 and GSE130567 accession codes.

Field-specific reporting

Please select the one below that is the best fit for your research. If you are not sure, read the appropriate sections before making your selection.

☒ Life sciences ☐ Behavioural & social sciences ☐ Ecological, evolutionary & environmental sciences

For a reference copy of the document with all sections, see nature.com/documents/nr-reporting-summary-flat.pdf

Life sciences study design

All studies must disclose on these points even when the disclosure is negative.

Sample size	No statistical methods were used to predetermine sample size. Sample size was chosen based on the literature and prior experience in our laboratory.
Data exclusions	No data was excluded from this study.
Replication	All experiments were performed as technical or biological replicates as appropriate for the experimental design and set-up.
Randomization	Random de-identified blood donor were used.
Blinding	Investigators were not blinded to experimental conditions.

Reporting for specific materials, systems and methods

We require information from authors about some types of materials, experimental systems and methods used in many studies. Here, indicate whether each material, system or method listed is relevant to your study. If you are not sure if a list item applies to your research, read the appropriate section before selecting a response.

Materials & experimental systems

n/a	Involved in the study
☐	☒ Antibodies
☒	☐ Eukaryotic cell lines
☒	☐ Palaeontology
☒	☐ Animals and other organisms
☒	☐ Human research participants
☒	☐ Clinical data

Methods

n/a	Involved in the study
☐	☒ ChIP-seq
☒	☐ Flow cytometry
☒	☐ MRI-based neuroimaging

Antibodies

Antibodies used

ChIP antibodies against
PU.1 (sc-352, SantaCruz)
C/EBPβ (sc-150, SantaCruz)
STAT3 (sc-482, SantaCruz)
c-Jun (sc-1694, SantaCruz)
p300 (sc-585, SantaCruz)
CDK8 (sc-1521,SantaCruz)
RNA Polymerase II (MMS-126R,Covance)
MED1 (A300-793A,Bethyl Laboratories)
SMC1 (A300-055A,Bethyl Laboratories)
Western Antibodies used:
STAT3 (Cell Signaling Technologies, Cat. No: 12640),
phospho-p38(Cell Signaling Technologies, Cat. No: 9215)
p38 (Cell Signaling Technologies, Cat. No: 9212)

Validation

All the above antibodies were per-validated by the corresponding vendors to work in either Western Blots or ChIP assays.

ChIP-seq

Data deposition

☒ Confirm that both raw and final processed data have been deposited in a public database such as GEO.

☒ Confirm that you have deposited or provided access to graph files (e.g. BED files) for the called peaks.

Data access links
May remain private before publication.

https://www.ncbi.nlm.nih.gov/geo/query/acc.cgi?acc=GSE120945
https://www.ncbi.nlm.nih.gov/geo/query/acc.cgi?acc=GSE130567

Files in database submission

ChIP-seq_CDK8_IFNg-LPS
ChIP-seq_CDK8_IFNg
ChIP-seq_CDK8_LPS
ChIP-seq_CDK8_Resting
ChIP-seq_CEBPB_IFNg-LPS_rep1

Resting1 RNA seq CD14+ Monocytes htseq-count read counts
Resting2 RNA seq CD14+ Monocytes htseq-count read counts
Resting3 RNA seq CD14+ Monocytes htseq-count read counts
LPS-16h-1 RNA seq CD14+ Monocytes htseq-count read counts
LPS-16h-2 RNA seq CD14+ Monocytes htseq-count read counts
LPS-16h-3 RNA seq CD14+ Monocytes htseq-count read counts
IFNG-3h-1 RNA seq CD14+ Monocytes htseq-count read counts
IFNG-3h-2 RNA seq CD14+ Monocytes htseq-count read counts
IFNG-3h-3 RNA seq CD14+ Monocytes htseq-count read counts
LPS-IFNG-1 RNA seq CD14+ Monocytes htseq-count read counts
LPS-IFNG-1 RNA seq CD14+ Monocytes htseq-count read counts
LPS-IFNG-1 RNA seq CD14+ Monocytes htseq-count read counts
D1-MoDC-GL RNA seq CD14+ Monocytes cultured in GM-CSF htseq-count read counts
D1-MoDC-G RNA seq CD14+ Monocytes cultured in GM-CSF htseq-count read counts
D1-MoDC-L RNA seq CD14+ Monocytes cultured in GM-CSF htseq-count read counts
D1-MoDC-R RNA seq CD14+ Monocytes cultured in GM-CSF htseq-count read counts
D2-MoDC-GL RNA seq CD14+ Monocytes cultured in GM-CSF htseq-count read counts
D2-MoDC-G RNA seq CD14+ Monocytes cultured in GM-CSF htseq-count read counts
D2-MoDC-L RNA seq CD14+ Monocytes cultured in GM-CSF htseq-count read counts
D2-MoDC-R RNA seq CD14+ Monocytes cultured in GM-CSF htseq-count read counts
D3-MoDC-GL RNA seq CD14+ Monocytes cultured in GM-CSF htseq-count read counts
D3-MoDC-G RNA seq CD14+ Monocytes cultured in GM-CSF htseq-count read counts
D3-MoDC-L RNA seq CD14+ Monocytes cultured in GM-CSF htseq-count read counts
D3-MoDC-R RNA seq CD14+ Monocytes cultured in GM-CSF htseq-count read counts
ChIP-seq_PU_1_INFg-LPS_rep2
ChIP-seq_SMC1_IFNg-LPS
ChIP-seq_SMC1_LPS
ChIP-seq_STAT3_IFNg_rep1
ChIP-seq_STAT3_IFNg_rep2
ChIP-seq_STAT3_IFNg-LPS_rep1
ChIP-seq_STAT3_IFNg-LPS_rep2
ChIP-seq_STAT3_IL10
ChIP-seq_STAT3_LPS_rep1
ChIP-seq_STAT3_LPS_rep2
ChIP-seq_STAT3_Resting_rep1
ChIP-seq_STAT3_Resting_rep2
ChIP-seq_CEBPB_IFNg-LPS_rep2
ChIP-seq_PolII_IFNg-LPS
ChIP-seq_PolII_LPS
ChIP-seq_PU_1_IFNg-LPS_rep1

Genome browser session
(e.g. UCSC)

"no longer applicable"

Methodology

Replicates

Because of limitations on cell numbers and to decrease variability related to differences among individual donors, chromatin immunoprecipitations were performed using pooled samples from more than two (for STAT3) or four (for CDK8 and SMC1) different donors. For STAT3, a second experiment with pooled samples from several donors was performed and congruence between the replicates was assessed by generating scatter plots and estimating Pearson correlation coefficients (Figure S4A). After ascertaining close correlation between replicates, we performed bioinformatic analysis using replicate 1 and confirmed key results using replicate 2.

Sequencing depth

A minimum of 20 million uniquely mapped reads were obtained for each condition.

Antibodies

ChIP antibodies against
PU.1 (sc-352, SantaCruz)
C/EBPβ (sc-150, SantaCruz)
STAT3 (sc-482, SantaCruz)
c-Jun (sc-1694, SantaCruz)
p300 (sc-585, SantaCruz)
CDK8 (sc-1521,SantaCruz)
RNA Polymerase II (MMS-126R,Covance)
MED1 (A300-793A,Bethyl Laboratories)
SMC1 (A300-055A,Bethyl Laboratories)

Peak calling parameters

We used the getDifferentialPeaksReplicates.pl with parameters -genome hg19 -balanced -t H3K27ac_L1/ H3K27ac_L2/ -b H3K27ac_R1/ H3K27ac_R2/ -p enhancers.bed from HOMER package and then merged using mergePeaks - size given.

Data quality

A false discovery rate (FDR) threshold of 0.001 was used for all data sets. Regions that overlap with blacklist identified by the ENCODE project were filtered out. The total number of mapped reads in each sample was normalized to ten million mapped reads.

Software	HOMER version 4.9.1 (Heinz et al., 2010)
