# Supplementary material for: IFN-γ selectively suppresses a subset of TLR4-activated genes and enhancers to potentiate macrophage activation
Source: Nat Commun. 2019 Jul 25;10:3320. doi: 10.1038/s41467-019-11147-3 (PMC6658531; doi:10.1038/s41467-019-11147-3)
Supplement: Supplementary file 3 — Description of Additional Supplementary Files [file 41467_2019_11147_MOESM3_ESM.pdf]

Description of Additional Supplementary Files

File Name: Supplementary Data 1

Description: **Gene Ontology (GO) analysis of genes enriched in each cluster (Figure 1d).** GO analysis was performed on genes in clusters I to VI using **D**atabase for **A**nnotation, **V**isualization and **I**ntegrated **D**iscovery (**DAVID**) v6.8 (https://david.ncifcrf.gov/) to identify enrichment of GO terms to understand the biological meaning behind the groups of genes in individual clusters. A list of enriched GO terms for each cluster shown in Figure 1d is provided. Highlighted in yellow are GO terms used in plotting the heat map (-log10 p-value) in Figure 1d.
